# The predictive value of early oral motor assessments for neurodevelopmental outcomes of moderately and late preterm infants

**DOI:** 10.1097/MD.0000000000009207

**Published:** 2017-12-15

**Authors:** Xianhong Zhang, Mei Zhou, Huaying Yin, Ying Dai, Yuwei Li

**Affiliations:** a Department of Neonatology; b Department of Primary Healthcare, Children's Hospital of Chongqing Medical University, Ministry of Education Key Laboratory of Child Development and Disorders; Key Laboratory of Pediatrics in Chongqing, Chongqing International Science and Technology Cooperation Center for Child Development and Disorders, Chongqing, China.

**Keywords:** assessment, moderately and late preterm, neurodevelopment, oral motor

## Abstract

Oral motor assessment is used to identify abnormal sucking patterns which may reflect neurodevelopmental problems in preterm infants, but few studies have focused on moderately and late preterm infants. We enrolled 118 moderately and late preterm infants (mean gestational age, 35.04 weeks; mean birth weight, 2347.59 g) and analyzed the relationship between the Neonatal Oral-Motor Assessment Scale scores of these infants and the Chinese revision of Bayley Scales of Infant Development outcomes at 6 months corrected age. And the infants with abnormal sucking pattern had significantly lower Mental Development Index and Psychomotor Development Index and showed a higher rate of below average scores than control group (*P* = .003, *P* = .029, *P* = .022). The incoordination of suck–swallow–respiration was a risk factor for adverse neurodevelopment (RR = 3.67, 95% CI: 1.42–9.45). These indicate that abnormal sucking patterns in moderately and late preterm infants might provide some predictive value for short-term neurodevelopmental outcomes, but the clinical predictive value for developmental delay need to be determined in a longer term follow-up. This finding may offer a basis for early intervention.

## 1 Introduction

With the development of neonatal–perinatal medicine, the survival rate of preterm infants has remarkably increased. Preterm infants born at <32 weeks of gestation are defined as very preterm (VPT) infants, and these infants have a high perinatal mortality and long-term developmental difficulties. At the same time, moderately and late preterm (MLPT, born by 32 0/7 to 36 6/7 weeks of gestation) infants are physiologically and developmentally immature at birth, and they make up the largest subgroup of preterm infants.^[1]^ Compared with VPT infants, MLPT infants have their particular physiological characteristics and underlying diseases. The physiological immaturity and various complications of MLPT infants may cause various types and degrees of brain injuries, influencing future neurodevelopment. Although apparent brain injuries are diagnosed by medical imageology including cranial ultrasound and magnetic resonance imaging, it is hard to identify preterm infants with no apparent neurobehavioral abnormalities in the early brain injury period.^[2]^

Studies have shown that sucking and swallowing disorders in early infancy serve as potential markers of neurodevelopmental problems, and abnormal sucking patterns may reflect brain developmental problems in preterm infants.^[3,4]^ In the past, MLPT infants have been considered to be at low risk for neurological impairments, so little attention has been paid to their development. However, increasing numbers of studies have reported that MLPT infants are at a greater risk for developmental disabilities and feeding problems than infants born at term.^[5]^ Previous researches have demonstrated that early sucking behavior assessment may predict neurological impairments of preterm infants, but there are few oral motor assessment studies that have focused on MLPT infants.^[6–8]^ At present, multiple tools have been developed over the years to evaluate sucking behaviors in neonates. The Neonatal Oral-Motor Assessment Scale (NOMAS) is a noninvasive neonatal feeding assessment tool that could be used in either bottle-feeding or breastfeeding and either preterm or full-term infants.^[9]^ The NOMAS has been translated and revised into Chinese, and the psychometrics of the NOMAS have been established.^[10,11]^

In the present study, we applied the NOMAS to assess the sucking patterns of MLPT infants, investigating the relationship between abnormal sucking patterns and neurodevelopmental outcomes at 6 months corrected age (CA). The aim was to determine whether oral motor assessments in MLPT infants provide predictive value for short-term neurodevelopmental outcomes.

## 2 Methods

### 2.1 Subjects

This study was a prospective, longitudinal study on the developmental outcomes of 121 MLPT infants born by 32 0/7 to 36 6/7 weeks of gestation. Infants were recruited from the newborn nursery of the Children's Hospital of Chongqing Medical University between October 2013 and January 2015. Infants with the following conditions were excluded: intraventricular hemorrhage (IVH) (grade III or IV) or periventricular leukomalacia; known metabolic or genetic diseases; complex congenital heart disease; congenital orofacial dysmorphism; congenital digestive tract malformation: for example, esophagus and rectum atresia, congenital megacolon; and serious infections: for example, purulent meningitis. This study was approved by the Institutional Review Board of Children's Hospital, Chongqing Medical University, China (approved no. 022/2011). And this study was registered in the Chinese Clinical Trial Registry (no. ChiCTR-ROC-17011195). Informed consent was obtained from the parents of all infants participating in this study.

A total of 121 infants were enrolled in this study, however, 2 infants withdrew and 1 infant missed the 6-month follow-up test, leaving 118 infants in this study. Among these infants, 13 infants were small for gestational age (SGA), 24 infants had low-grade IVH, and 65 infants were low birth weight (LBW).

### 2.2 NOMAS

The NOMAS, first reported by Braun and Palmer^[12]^ and developed by Palmer et al^[13]^, is a diagnostic tool for neonatal feeding difficulty.^[12,13]^ It includes a 28-item checklist of jaw and tongue movement. The infants were classified into a normal sucking pattern, a disorganized sucking pattern, or a dysfunctional sucking pattern, according to the NOMAS assessment. A normal sucking pattern is defined as a newborn who has attained coordination of suck–swallow–breath in both non-nutritive and nutritive sucking, and the ratio is 1:1:1 and 10 to 30 sucks in a burst.^[10]^ A disorganized sucking pattern is defined as a newborn who is unable to sustain coordination of suck–swallow–breath or has a lack of rhythm in movement.^[14]^ A dysfunctional sucking pattern is defined as a newborn whose motor reactions as well as jaw and tongue movements are abnormal or there is no sucking movement.^[10]^

In this study we referred to the scoring system Case-Smith et al^[15]^ reported. For a normal sucking pattern, the assessor assessed the newborn and rated them on a 3 scale: 0, not at all; 1, <50% of the time, and 2, >50% of the time. Normal sucking pattern scores ranged from 1 to 20. The higher score they gained the better sucking function they were. For a disorganized and dysfunctional sucking pattern, a score of 1 if they were made as present and a score of 0 if they were made as absent. The scores ranged from 1 to 10 of the disorganized and dysfunctional sucking patterns. The lower score they gained the better sucking function they were.

### 2.3 NOMAS evaluation procedure

According to Palmer's method, the bottle-feeding behavior of preterm infants was recorded by a digital camera once a week before they were discharged. The videos were numbered by a researcher who did not participate in the assessment process. Two assessors who had been trained by NOMAS courses assessed the first 2-min episode of sucking behavior blindly in order to distinguish the infant's sucking pattern. For each episode, the infant was scored as having a normal, disorganized, or dysfunctional sucking pattern. Based on the NOMAS scores at 36- to 37-week postmenstrual age (PMA), infants who were scored as having a normal sucking pattern were assigned to the normal sucking pattern group and infants who were scored as having a disorganized or dysfunctional sucking pattern were assigned to the abnormal sucking pattern group. If the result of grouping was different, 2 assessors discussed the video until consensus was reached. The inter-rater reliability between the 2 assessors was 0.841 before the discussion.

## 3 Follow-up at 6 months CA

### 3.1 Anthropometry measurements

Anthropometry measurements including weight, supine length, and head circumference were recorded for each subject at 6 months CA. All measurements were obtained by nurses of child health care according to standardized techniques using calibrated equipment. Weight was recorded as 0.1 kg; supine length and head circumference were recorded as 0.1 cm, respectively. And doctor finished regular physical examination and the feeding guide.

### 3.2 Assessment of infants neurodevelopment

We used the Chinese revision of the Bayley Scales of Infant Development (BSID-CR) to evaluate the neurodevelopment outcomes of infants. The split-half related coefficient of the BSID-CR was 0.69 to 0.98, and the inter-rater reliability was 0.86. We applied the BSID-CR to assess the infant's cognition, language, gross, and fine motor skills. The raw score was transformed as the mental development index (MDI) and the psychomotor development index (PDI). A BSID-CR index score with the MDI or PDI <90 was classified as below average, and <80 was classified as being in a critical state. At 6 months CA, the BSID-CR tests of the infants were completed by a psychologist.

### 3.3 Statistical analysis

The data were analyzed by the statistical software package SPSS V.19.0 (SPSS Inc, Chicago, IL). *P* < .05 was considered to be statistically significant. The Mann–Whitney *U* test, independent samples *t*-test, and Pearson's χ^2^ analysis were used to compare differences of sample characteristics between the 2 groups. The independent samples *t*-test was used to determine the differences in nutritional status, and BSID-CR scores between groups at 6 months CA, and the predictive value was investigated using Pearson's χ^2^ analysis and Fisher exact test to determine the rate of developmental delay related to abnormal sucking patterns. Relative risks (RRs) and 95% confidence intervals (CIs) of each item were calculated to determine the risk of abnormal sucking patterns on adverse neurological development.

## 4 Results

According to the NOMAS scores at 36 to 37 weeks PMA, 68 infants (41 males, 27 females) were assigned to the normal sucking pattern group, and 50 infants (27 males, 23 females) were classified as having a disorganized sucking pattern and were assigned to the abnormal sucking pattern group (see Table 1). Because our sample did not enroll infants with neurological disorders or digestive organic disease, no infants were classified as having a dysfunctional sucking pattern. There were no statistical differences in gender (*P* = .494), gestational age (*P* = .068), birth weight (*P* = .856), Apgar score (*P* = .061), SGA (*P* = .138), LBW (*P* = .864), IVH (*P* = .397), or length of stay (*P* = .138) between the groups.

**Table 1:**
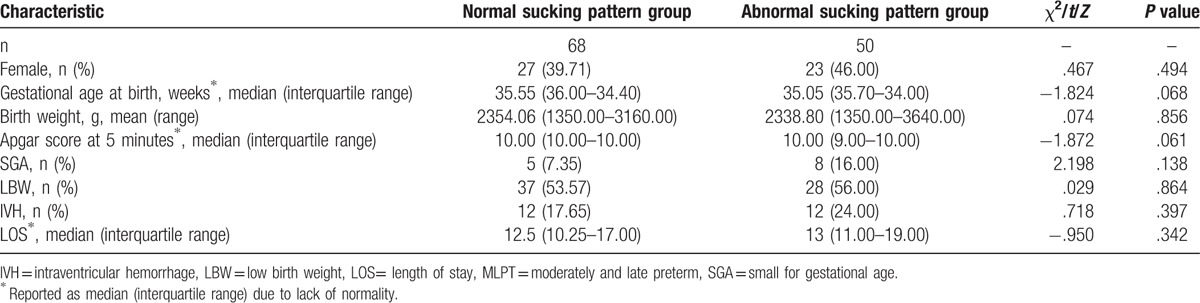
Characteristics of the MLPT infants.

The comparison of nutritional status between 2 groups at 6 months CA is presented in Table 2. There were no statistical differences in weight, supine length, or head circumference between 2 groups (*P* = .599, *P* = .967, *P* = .681).

**Table 2:**
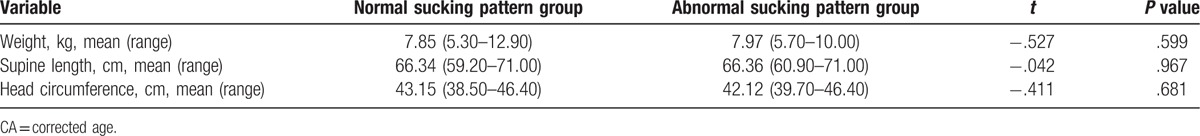
Comparison of postnatal nutritional status between the groups at 6 months CA.

The relationship between abnormal sucking patterns in MLPT infants and neurodevelopmental outcomes is shown in Table 3. At 6 months CA, the MLPT infants in the abnormal sucking pattern group had lower MDI and PDI scores than infants in the normal sucking pattern group (*P* = .003, *P* = .029). A total of 14% (n = 16) of the infants had a below average score (MDI or PDI <90), and 3% (n = 3) of the infants had a critical state score (MDI or PDI <80). Infants in the abnormal sucking pattern group showed a higher rate of below average scores than the normal sucking pattern group (*P* = .022). There was no significant difference in the rate of critical state score between the groups (*P* = .573).

**Table 3:**
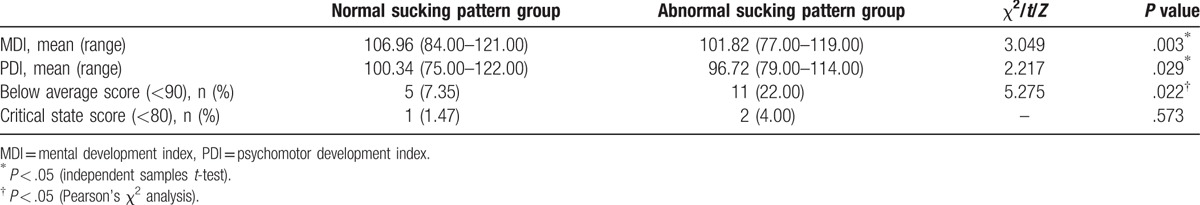
Relationship between abnormal sucking patterns and neurodevelopmental outcomes.

RRs and 95% CIs of NOMAS items for adverse neurodevelopmental outcomes are shown in Table 4. The arrhythmical jaw and tongue movements, difficulty initiating movements, and incoordination of suck/swallow and respiration were risk factors for adverse neurodevelopment (RR >1). Among these items, incoordination of suck/swallow and respiration reached significance (RR = 3.67, 95% CI: 1.42–9.45).

**Table 4:**
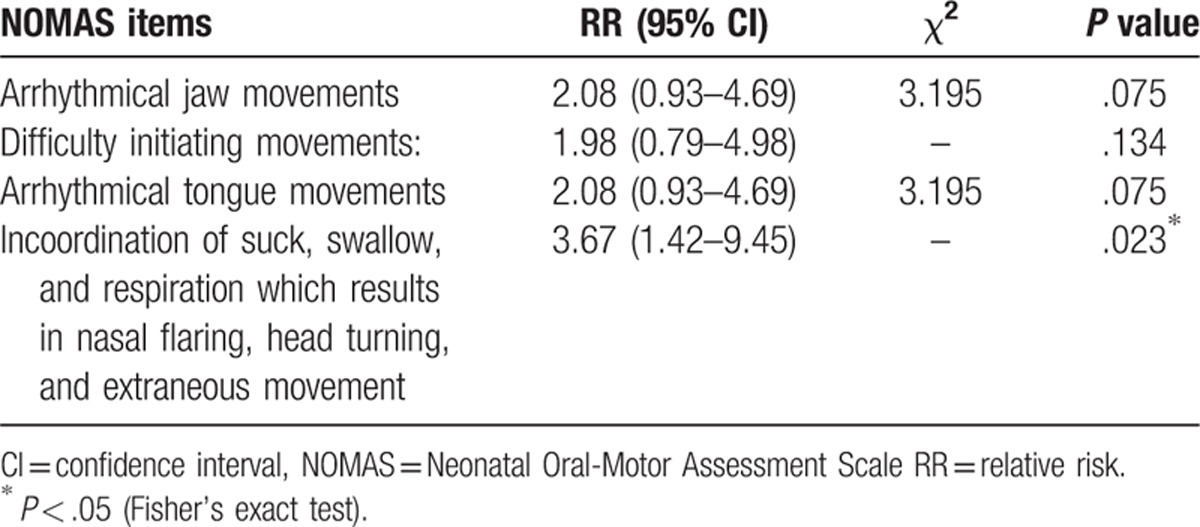
RRs and 95% CIs of NOMAS items for adverse neurodevelopmental outcomes.

## 5 Discussion

Our study demonstrates that abnormal sucking patterns in MLPT infants are related to adverse neurodevelopmental outcomes at 6 months CA. And incoordination of sucking, swallowing, and breathing may be an early signal of adverse neurodevelopmental outcomes. These results indicate that abnormal sucking patterns show some predictive value on short-term neurodevelopmental outcomes, but the predictive value for developmental delay needs to be determined in a longer term follow-up study.

It is known that an infant's brain injury in the neonatal period has an adverse influence on neurodevelopmental outcomes. Nutritional status has also been shown to be related to development. In the present study, no differences were found in weight, supine length, or head circumference between the groups. These results suggest that we eliminated the effect of postnatal nutritional status on the BSID-CR scores of MLPT infants.

Sucking is a basic activity of newborns that is controlled by central pattern generators in the brain.^[16]^ Sucking has been seen in the fetus as early as 15 weeks of gestation.^[17]^ An immature suck–swallow pattern occurs at 32 to 34 weeks of gestation, and the pattern consists of swallowing before or after short sucking bursts. The mature suck–swallow pattern is gradually built after 34 weeks’ gestational age, and the ratio of these actions is 1:1.^[18]^ Successful and safe oral feeding depends on the coordination of suck–swallow–breath and brain development of the infant.^[19]^ Full-term infants acquire this coordination at birth. Because the bulbar central pattern generators for coordination of suck–swallow–breath in preterm infants are immature, preterm cannot attain it until term age.^[20]^ Due to these characteristics of preterm development, the NOMAS score at 36 to 37 weeks PMA was chosen for sucking pattern identification in our study. Previous studies have noted that the rhythmic processes involved in sucking are under bulbar control, especially in the regions of the nuclei ambiguus, solitarius, and hypoglossus in the lower medulla. Efferent and afferent cranial nerves (V, VII, XI, X, and XII) are involved in the control of mastication, respiration, and swallowing.^[19]^ Moreover, the relative volume of myelinated white matter during 36 to 41 weeks of gestation increased 5-fold, compared to that at 29 to 34 weeks of gestation.^[21]^ These evidences show that nervous system development and maturity of sucking organization occur in parallel.

Few studies have investigated prediction of oral motor assessment for neurodevelopment in MLPT infants. In the present study, we used the NOMAS as an evaluation standard to identify the sucking patterns of MLPT infants and obtained a larger and more specific sample than previous studies. Palmer and Heyman^[3]^ first applied the NOMAS to examine the correlation between the sucking pattern and later development in a small population. The follow-up study enrolled 34 subjects in total, and 18 subjects were examined. Of 16 infants who had a disorganized or dysfunctional sucking pattern, 11 preterm infants showed developmental delay at 24 months. Tsai et al^[8]^ assessed 27 preterm infants without brain injury at weekly intervals with the NOMAS, and infants with persistent disorganized sucking patterns after 37 weeks PMA had lower BSID scores than the control group at 6 and 12 months CA. Our results are in line with these similar studies. However, in the study by Tsai et al, the rate of developmental delay between the 2 groups was significantly different, which we did not find in our study. Actually, there were 3 infants who had developmental delay at 6 months CA, and the rate of developmental delay was lower than that reported by Tsai et al's study (2.54% vs 29.73%). In addition, Schonhaut et al^[22]^ noted that there was a linear inverse relationship between weeks of the gestational age and the risk of developmental delay. The average gestational age in our study was 35.04 ± 1.13 weeks (range, 32.00–36.90 weeks), which is older than that in the aforementioned studies. Furthermore, Reuner et al^[23]^ analyzed cognitive development in extremely/very preterm to moderately/late preterm and full-term infants and found that the extremely/very preterm group had the lowest focused attention, indicating the highest impact of prematurity in the most immature infants. These might possibly explain the discrepancy.

Moreover, a detailed analysis of specific items of the NOMAS seems to be important. Analysis of the RR of NOMAS items on neurodevelopment indicated that infants exhibited abnormal specific items including arrhythmical jaw and tongue movements, difficulty initiating movements, and incoordination of suck–swallow–respiration were higher risk on adverse neurodevelopment at 6 months CA. Among these items, incoordination of suck–swallow–respiration reached significance. Coordination of suck–swallow–respiration is attained when infant can take oral feedings with no episodes of desaturation, apnea, bradycardia, or aspiration and demonstrate a ratio of 1:1:1 or 2:2:1.^[24]^ This ability involve in the improvement of the brain structure functions including mature of cranial nerve, brain stem area, and cortex area. The delay in attaining coordination may be a signal of neurodevelopmental abnormalities. Early identification of premature infants with abnormal specific sucking features is helpful for providing early strategies.

In recent research, Wolthuis-Stigter et al^[25]^ reported that arrhythmic jaw and tongue movements, the incoordination of suck–swallow–respiration, the inability to sustain the sucking pattern, and the absence of a mature sucking pattern beyond an appropriate age increased the odds of abnormal neurodevelopmental outcomes at an age of 2 years. Our study is similar to the Wolthuis-Stigter et al's research, but the age of follow-up is 6 months CA which is younger than Wolthuis-Stigter et al's study. In the actual process of evaluation, we observed that a continuous sucking phase of most premature infants did not last 2 minutes. Mizuno and Ueda^[7]^ noted that in the case of preterm infants (approximately until full-term age), the continuous phase only lasts about 30 seconds, influenced by neurological function and cardiorespiratory control. So it seems that unable to sustain suck pattern for 2 minutes and persistence of immature suck pattern beyond an appropriate age are not realistic to apply in preterm infants before they have reached 40 PMA. Due to the age of assessment was <40 PMA in our study, few infants showed above 2 abnormal sucking features, resulting in these sucking features were not classified as risk factors (RR <1). However, as an infant gestation increased, the sucking skill would mature. We assume that the age at which the sucking behavior is assessed is related to the sensitivity of specific elements of abnormal sucking pattern, thus, further research is required to explore this relationship.

In our research, infants in the abnormal sucking pattern group showed a higher rate of below average scores than the normal sucking pattern group at 6 months CA, and the age at follow-up was younger than that of most similar researches. These findings suggest that the clinical predictive value of abnormal sucking patterns for developmental delay in MLPT infants is limited in the short term. However, the MLPT infants with abnormal sucking patterns were also at a higher risk for adverse neurodevelopmental outcomes than the control group. We are unable to neglect the infants who have a below-average score of developmental outcomes. Moreover, brain development is complicated and influenced by external factors such as parental education and environment. Therefore, a longer term follow-up for this population is needed. In addition, we suggest that more attention should be given to MLPT infants with abnormal sucking patterns. The brain has a compensatory and restructuring ability in the early period, and sensation stimuli including visual, sound, and touch from the environment can promote some parts of the brain to generate new neurons that may replace damaged neurons, which can compensate brain function. Based on the characteristics of brain development, providing timely and effective interventions for MLPT infants with abnormal sucking patterns may improve developmental outcomes.

There were several limitations in our study. First, some parts of the NOMAS are based on subjective judgments of the assessor which may relatively lack objectivity. Second, because the maturity of the sucking behavior is closely correlated with the PMA, the sucking pattern should be evaluated in succession from birth to term-equivalent age. However, our study lacked the longitudinal developmental trajectory of the sucking pattern. Lastly, none of the infants in our sample were classified as having a dysfunctional sucking pattern and the follow-up period was short.

In conclusion, abnormal sucking patterns in MLPT infants might provide some predictive value for short-term neurodevelopmental outcomes, and a longer term follow-up is needed to investigate their clinical prognostic value for later developmental outcomes. In addition, incoordination of sucking, swallowing, and breathing may be an early signal of adverse neurodevelopmental outcomes. Which specific elements of abnormal sucking pattern are more sensitive at predicting abnormal neurodevelopment need to be further explored in future studies. Due to the fact that the brain has strong plasticity in the early postnatal period, our study suggests that early intervention may be necessary for these infants.
